# Neuroprotective effect of *Spirulina fusiform* and amantadine in the 6-OHDA induced Parkinsonism in rats

**DOI:** 10.1186/s12906-015-0815-0

**Published:** 2015-08-25

**Authors:** I Chattopadhyaya, Sumeet Gupta, Asad Mohammed, N Mushtaq, S Chauhan, Saikant Ghosh

**Affiliations:** Department of Pharmacology, M. M. College of Pharmacy, M. M. University, Mullana, (Ambala), Haryana India; College of Applied Medical Sciences, Shaqra University, Shaqra, Saudi Arabia; FDD-NDDS Lipid Research Group, Sun Pharma Advance Research Centre, Vadodra, Gujarat, India

**Keywords:** Anti-oxidant assays, Dopamine level, Rotational behavior, Spirulina

## Abstract

**Background:**

Multi-factorial etiology exists in pathophysiology of neurodegenerative diseases. The imbalance of anti-oxidant enzymes and dopamine level leads to Parkinsonism. The objective of this study was to assess the protective effect of *Spirulina fusiform* alone and in combination with amantadine against Parkinsonism effect in 6-hydroxydopamine (6-OHDA) induced rat model.

**Methods:**

*S. fusiform* was administered in different groups (500 mg/kg, once daily and twice daily) and a combination of spirulina (500 mg/kg, once daily) with amantadine (20 mg/kg once daily) for 30 days before and 14 days after a single injection of 6-OHDA into the dorsal striatum. Post lesion produced rotational behavior which was measured at two week intervals (37^th^ and 44^th^ day). Locomotors activity was also done at 44^th^ and muscle coordination at 48^th^ day. Dorsal striatum was isolated from rat brain for evaluating the antioxidant assays and dopamine content at 49^th^ day.

**Results:**

Both the body rotations (ipsilateral and contralateral) were found to have a statistically significant (*p < 0.001*) decrease by 34.26 and 52 % after treatment with spirulina (Twice a day) in spirulina treated lesioned group. A higher percentage of improvement was shown in the reduction of ipsilateral (57.34 %) and contralateral (78.3 %) rotations in combination of spirulina with amantadine treated lesioned group rather than spirulina alone treated lesioned groups when compared with positive control lesioned group. Body movements and locomotor activity were improved statistically *(p < 0.0001)* significant in both treated lesioned groups (Combination of spirulina with amantadine and spirulina twice daily). Similar results were also seen in anti-oxidant levels which later on reached to the normal value. The levels of dopamine content had a statistically significant (*p < 0.0001*) increase by 78.3 % only in case of spirulina with amantadine treated lesioned group.

**Conclusion:**

Spirulina is a potent nutraceutical supplement all over the world, so my preclinical study may contribute to give an additional adjuvant drug therapy in aging related disorders (Neurodegenerative as well as diabetes associated neurodegenerative disorders).

**Electronic supplementary material:**

The online version of this article (doi:10.1186/s12906-015-0815-0) contains supplementary material, which is available to authorized users.

## Background

Neurodegenerative disorders encompass a condition where nerve cells of different parts of brain and spinal cord vanish leading to either functional loss or sensory dysfunction [1]. Parkinson’s disease (PD) is second most prevalent neurological disorder that is characterized by progressive neurodegeneration affecting dopaminergic neurons in the negrostriatal pathway [2]. The most common symptoms of Parkinsonism are akinesia, bradykinesia, rigidity, tremor and postural abnormalities [3, 4]. Some of the common causes which are responsible for etiology of this disorder include oxidative stress, mitochondrial damage, protein dysfunctions, and inflammation [1]. Pathologically, Parkinson’s disease is characterized by the significant degeneration of dopaminergic neurons in the *substantia nigra pars compacta*, which leads to the depletion of dopamine in its striatal projections and other brain stem neurons, with consequent disruption of the cerebral neuronal systems responsible for motor functions. Despite of major advances in the current understanding of Parkinsonism pathology, the exact mechanism involved in this neurodegenerative cascade remains unclear. However, many studies claim that excessive generation of reactive oxygen species (ROS) and inflammation may play a central role in the neuropathology of PD [5].

Some of the studies suggest the use of nutraceuticals as natural antioxidants as an appropriate strategy to diminish the progression of neurodegenerative diseases [6]. The nutraceuticals can be given as an adjuvant therapy to fabricate synergistic/additive effects and to reduce the dose of drug.

Spirulina *(Spirulina fusiform)*, a type of blue green algae, is a cyanobacterium belonging to the Oscillatoraceae family. It is gaining more attention by medical scientists as a nutraceutical and as a source of potential pharmaceutical. It has been consumed as primary food source by Aztecs and Mayans since thousands of years. Recently, we also reported the positive interaction between spirulina and glitazone to prevent diabetic osteoporosis [7–9]. Several studies revealed the potential properties of spirulina include anti-carcinogenic, immunostimulants, antigenotoxic, anti-hepatotoxic, anti-inflammatory, anti-diabetic and anti-hypertensive. Spirulina is also well known as anti-oxidant nutraceutical [10] as it contains high levels of antioxidant and anti-inflammatory molecules such as c-phycocyanin, one of the richest sources of plant proteins (60–70 %), vitamins especially vitamin B_12_ and provitamin A (β-carotene), γ-linolenic acid, phenolic compounds and minerals like calcium, chromium and magnesium [11, 12]. Another species, *Spirulina platensis* extract attributed protective response on birds, aquatic and animal’s species against oxidative stress. Studies have shown that chronic treatment of spirulina demonstrated a potent free radical scavenging activity on *Oreochromis niloticus* [13] and albino wistar rats after exposed to deltamethrin intoxication [14]. The protective effect of spirulina against hepatotoxicity caused by erythromycin thiocyanante induced oxidative damage on Egyptian baladi bucks reported by another researcher [15]. Spirulina shows significant immunodulatory effect in acetic induced colitis in experimental rats [16].

Number of studies has shown spirulina to exert protective effect in the treatment of neurodegenerative disorders (Parkinsonism and Alzheimer disorder). Stromberg study also suggests a protective response by enhancement in the recovery of striatal dopamine tyrosine hydroxylase (TH) positive fibers and TH positive neurons in the *substantia nigra* pars compacta in experimental rat induced Parkinsonism after feeding with spirulina [17]. Spirulina exerts a significant protective effect on hippocampus neural progenitor cells against lipopolysaccharide induced acute systemic inflammatory [18]. Anti-inflammatory and antioxidant effect of spirulina demonstrate a neuroprotectivity against adeno-associated virus vector (AAV9) in *substantia nigra* of rats reported by Prabon [19]. Amantadine is an antiviral drug that is also used in the treatment of PD. Basically; it is a synthetic compound which can be used in advanced stage of Parkinsonism and motor fluctuations. However, this drug has a very fewer side effects other than anti Parkinsonism drugs. So, it can be used at low doses for chronic treatment [20]. Though, many literatures suggest that spirulina is a strong supplementation food and reported as anti-oxidant compound for the prevention of Parkinsonism but it could be used in combination with amantadine for the pretreatment of Parkinsonism on rats. Hence, the present study was undertaken to assess the neuroprotectivity of spirulina alone and in combination with amantadine in experimental rat induced Parkinsonism.

## Methods

### Drugs and chemicals

6-hydroxy dopamine hydrochloride (6-OHDA HCL), amantadine, apomorphine hydrochloride, thiobarbituric acid (TBA) and ascorbic acid were purchased from Sigma Aldrich lab ltd, (Foreign Holding Chemical Company, Bangalore, India). All other reagents and chemicals used in the study were of HPLC analytical grade. *Spirulina fusiformis* in the form of powder was a gift from RECON Ltd., (Bangalore, India).

## Animals

Adult male wistar albino rats were supplied by National Institute of Pharmaceutical Education Research, (Mohali), Punjab. At the beginning of the study, the rats aged were 7–8 weeks old and weighed about 180–200 g. Before the surgical procedures, the rats were housed in stainless steel cages and kept on a 12 h light/dark cycle (lights on at 7.30 a.m, lights off from 7.30 p.m.) at an ambient temperature of 24 ± 2 °C. Animals had free access to pelleted food (Lipton India, Ltd) and water a*d-libitum*. All experiments were performed according to the regulations and with prior approval from Institute Animal Ethics Committee (Reg. no. MMCP/IAEC/11/22).

## Preparation of *Spirulina fusiform*

The spirulina powder (50 gm) was soaked in 1 L of ultra-pure water and shaken continuously for 24 h at room temperature. The mixture was then centrifuged at 5,000 rpm for 10 min (4 °C) and the supernatant was filtered (Whatmann No. 1) to remove the cell debris. The sample was then freeze-dried and the dried extract was stored at 4 °C before use for the experiments. Freeze-dried extract was suspended in vehicle (olive oil) and was given to each rat by oral gavage daily, for 30 days prior to lessioning. The extract was administered continuously for 14 more days after conducted the surgery at the same day with same dose.

### Experimental design

#### 6-OHDA-induced Parkinsonism

Animals were divided into eight groups. Each group consisted of 14 animals

- Group 1: Vehicle treated sham operated control (NS)
- Group 2: *Spirulina fusiform* (500 mg/kg bodyweight/day per oral) treated sham operated group (SS)
- Group 3: *Spirulina fusiform* (500 mg/kg bodyweight/twice a day per oral) treated sham operated group (STS)
- Group 4: Vehicle treated lesioned control—lesions induced using 2 μL of 6-OHDA in ascorbic acid-saline (LPC)
- Group 5: Amantadine 20 mg/kg bodyweight treated lesioned group (LA)
- Group 6: *Spirulina fusiform* (500 mg/kg bodyweight/day per oral) treated lesioned group (LS)
- Group 7: *Spirulina fusiform* (500 mg/kg bodyweight/twice a day per oral) treated lesioned group (LST)
- Group 8: *Spirulina fusiform* (500 mg/kg bodyweight/day per oral) + Amantadine 20 mg/kg orally treated lesioned group (LSA)

### Experimental Parkinsonism

Pretreatment with spirulina for continuously 30 days, thirty minutes prior to surgery on day 31, rats received *i.p*. 15 mg/kg of desipramine hydrochloride in order to inhibit 6-OHDA-induced degeneration of noradrenergic pathways [21]. Then, rats were lightly anaesthetized with sodium pentobarbitone (45 mg/kg bodyweight, *i.p*) and were placed in a stereotaxic apparatus. A stainless steel needle (0.28 mm o.d) was inserted unilaterally through a small hole in the skull and the needle tip was placed into the dorsal striatum. The stereotaxic adjusted according to the atlas of paxinos and Watson (1986) and was follows as: A/P = −2.8 mm, L = +1.8 mm, D/V = −8.6 mm [22]. Before surgery, freshly prepared solution of 6 −hydroxydopamine (6-OHDA) hydrochloride at a dose of 10 μg dissolved in a 4 μl of sterile 0.9 % NaCl supplemented with 0.05 % ascorbic acid and was slowly infused into the dorsal striatum at a flow rate of 0.5 μl/min using a 10-μl Hamilton syringe. After entire administered of 6-OHDA, the cannula was left in place for further 5 mins for complete diffusion of the toxin and then was slowly retracted. Sham-operated rats were also treated in the same manner, but received equivalent volumes of vehicle instead of 6-OHDA hydrochloride.

### Post-operative care

The effect of anesthesia was recovered by approximately 4–5 h. The rats were kept in a well-ventilated room at 25 ± 3 °C in individual cages, till they gained full consciousness and then were housed together in a group of four animals per cage. Food and water was kept inside the cages for the first week so that animals could easily access it without any physical trauma due to overhead injury.

### Behavioral parameters

All behavioral analysis was performed by an observer blinded to the group. Different tests were performed at different time points after lesion over a period of 3 weeks.

### Amphetamine-induced rotations

Amphetamine-induced rotations [23] were performed on 37^th^ day after administering 6-OHDA injection to evaluate the extent of dopaminergic lesion. Rats were received d-amphetamine sulfate (5.0 mg/kg *i.p)* and were placed in rotometer bowls. They were allowed to habituate in their environment for 10 min before turns circling in ipsiversive directions to the lesion and were recorded continuously for 90 min. Results were expressed in ipsilateral net turns/5 min.

### Apomorphine-induced rotations

Apomorphine-induced rotations [23] were performed on 44^th^ day at post-lesion. Apomorphine was injected *s.c* at a dose of 0.5 mg/kg and rotations were monitored using the same experimental setup as for amphetamine-induced rotations. Results were expressed as contralateral net turns/5 min [5].

### Spontaneous motor test

#### Locomotor activity

On 44^th^ day, all the rats were tested for locomotor activity in animal activity chamber [5]. The rats were placed in the chamber individually and its locomotor activity was monitored visually. The activity chamber was furnished with black paper for easily assessing the different parameters include locomotion, rest, rearing, stereo events (number), rotations (clockwise), rotations (anti clockwise) and distance travelled (cm). The activity chamber was swabbed with 10 % alcohol at every time to avoid the interference due to rat odors.

### Rota rod (muscular coordination)

The muscular coordination was determined at 48^th^ day using digital rota rod apparatus. It consists of rotating rod having 75 mm diameter and were divided into four parts by compartmentalization to permit the testing for individual rats at a same time [5]. The apparatus automatically records the time in seconds when the rats fall from the rotating shaft. The speed was set at 10 r.p.m and cut of time was 180 s.

### Biochemical studies

#### Tissue preparation for antioxidant assays

On 49^th^ day, six rats were sacrificed from each group and their brains were isolated quickly for harvesting striatum and substantia nigra by cutting acrononal section of 1.0 mm thickness using rat brain matrix in the light of rat brain atlas [22]. *Substantia nigra* was used for the estimation of thiobarbituric reactive substances (TBARS) and reduced glutathione. Striatum was used for dopamine levels.

### Assay for thiobarbituric reactive substances, a marker of lipid peroxidation

Estimation of lipid peroxidation was investigated by Utley method [24]. About, 0.2 mL of post-mitochondrial supernant liquid was pipetted in an eppendorf tube and incubated at 37 ± 1 °C in a metabolic water bath shaker for 60 min at 120 strokes up and down. Another 0.2 mL was also pipetted in an eppendorf tube and placed at 0 °C for incubation. After 1 h of incubation, 0.4 mL of 5 % trichloro acetic acid and 0.4 mL of 0.67 % thiobarbituric acid were added in both the samples (i.e. 0 °C and 37 °C). The reaction mixture was then transferred from the vial to the tube and centrifuged at 1125 g for 15 min. The supernatant was transferred to another tube and placed in a boiling water bath for 10 min. Thereafter, the test tubes were cooled and the absorbance was measured at 535 nm. The rate of lipid peroxidation was expressed as nmol of TBARS reactive substance formed/(h mg protein).

### Assay for reduced glutathione content

Reduced glutathione was determined by the method of Jollow, 1974 [25]. About 0.2 mL of post-mitochondrial supernant liquid (10 % *w*/*v*) was precipitated with 0.2 mL of sulfosalicylic acid (4 %). The sample were kept at 4 °C for at least 1 h and then subjected to centrifugation at 1200 g for 15 min at 4 °C. The total volume (2 mL) of assay mixture contained 0.1 mL of filtered aliquot sample (10 % *w*/*v*), 1.7 mL of phosphate buffer (0.1 M, pH 7.4) and 0.2 mL of 5-thio-2-nitrobenzoic acid (4 mg/1 mL of phosphate buffer, 0.1 M, pH 7.4). After yellow color developed, it was measured immediately at 412 nm.

### Estimation of dopamine levels

Isolated dorsal striatum were weighed, frozen immediately in liquid nitrogen and stored at −70 °C until the sample preparation. After thawing, tissue samples were homogenized (1:10 *w*/*v*) in 0.1 M perchloric acid with ultrasonic disintegrator. The homogenates were centrifuged for 15 mins at 15000 g at 4 °C, supernatants were filtered and diluted (1:1) with 0.1 M perchloric acid. Dopamine content in tissue homogenates of striatum were determined by High-performance Liquid Chromatography (HPLC) with subsequent electrochemical detection method [26–28] for separation and quantification. The column was maintained at 35 °C. The mobile phase consisted of 0.1 M potassium phosphate (pH 4.0), 10 % methanol, and 1.0 mM heptanes sulfonic acid. Samples were separated on an ODS-C18 column using a flow rate of 1.0 mL/min. The concentrations of DA were calculated using a standard curve generated by determining ratio between known amounts of the amine and a constant amount of internal standard and represented as ng/mg of tissue.

### Statistical analysis

The data were expressed as mean ± Standard deviation. The statistical significances between means were analyzed using One-way analysis of variance (ANOVA) followed by Tukey’s multiple range test. A *p* < 0.05 was considered as statistical significant. Multivariate analysis was done with post hoc bonferri test with SPSS Inc 16.0 software.

## Results

### Effect of Parkinsonism on behavior activity and its restoration by *Spirulina fusiformis*

**Fig 1:**
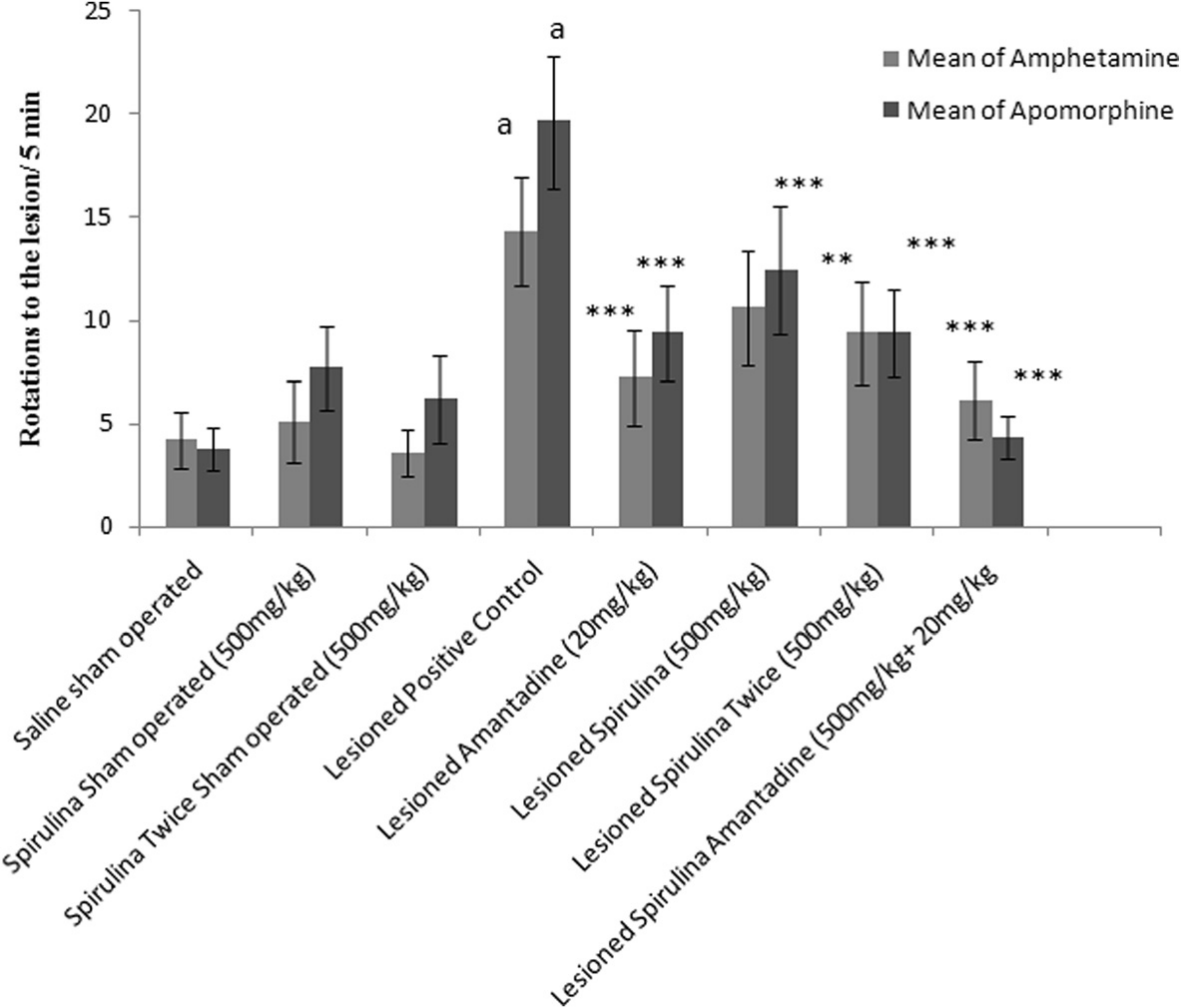
The effect of treatment on body rotations. Each bar represents the mean ± standard deviation of six animals and the experiment were repeated twice. ^a^ p < 0.001 vs. Saline sham operated, *p < 0.05,**p < 0.01,***p < 0.001 VS Lesioned positive control operated

After post lesion, at 37^th^ day, ipsilateral body rotations were increased by 70.62 % with amphetamine and 80.62 % contra lateral body rotations were produced by apomorphine on 44^th^ day in the lesioned positive control group (Fig. 1) when compared with normal saline sham operated group. Thirty days prior and 14 days after the administration of 6-OHDA, twice daily dose of spirulina treated lesioned group showed a good statistically significant (*p < 0.001*) reduction in ispilateral circling behavior by 34.26 and 52 % were decreased in contra lateral body rotations. Overall, on 37^th^ day, spirulina treated lesioned groups and in combination of spirulina with amantadine treated lesioned group showed a statistically significant decline in number of rotations as per this order (LSA>LA>LST>LS) and on 44^th^ day, the effect was shown like this LSA>LA = LST>LS.

A positive statistical (*p < 0.0001*) significantly interaction was found in each group between behavior-behavior interaction (Additional file 1: Table S1) after induced by amphetamine and apomorphine. With post hoc analysis, among all groups, a positive lesioned control group (Group 4; Additional file 1: Table S1) showed a statistically significant (*p < 0.0001*) difference in their results when compared to all groups.

**Fig. 2:**
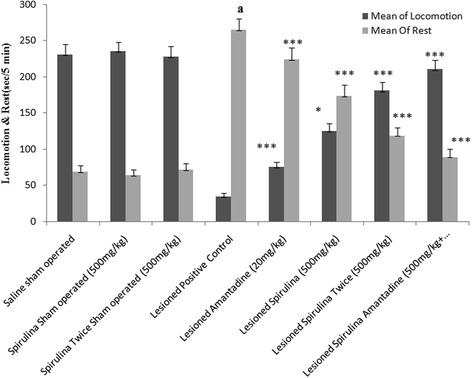
The effect of treatments on body locomotion & rest. Each bar represents the mean ± standard deviation of six animals and the experiments were repeated twice. ^a^ p < 0.001 vs Saline sham operated, *p < 0.05,**p < 0.01,***p < 0.001 VS Lesioned positive control operated

In activity chamber, lesioned positive control group showed a significantly declined in percentage (73.81 %) of locomotion activity when compared with normal saline sham operated group (Fig. 2). A dose dependent effect was shown by daily administration of single dose of spirulina (71.6 %) and twice dose of spirulina (80.6 %) treated lesioned group demonstrated a significant improvement in their body movements and extremely highly statistical (*p < 0.0001*) significant difference in their results on 44^th^ day. A high percentage (83.4 %) of locomotors activity was obtained after treatment with spirulina plus amantadine treated lesioned group demonstrated a highly statistically significant (*p < 0.0001*) difference as mentioned in body movement and rest activity.

**Fig. 3:**
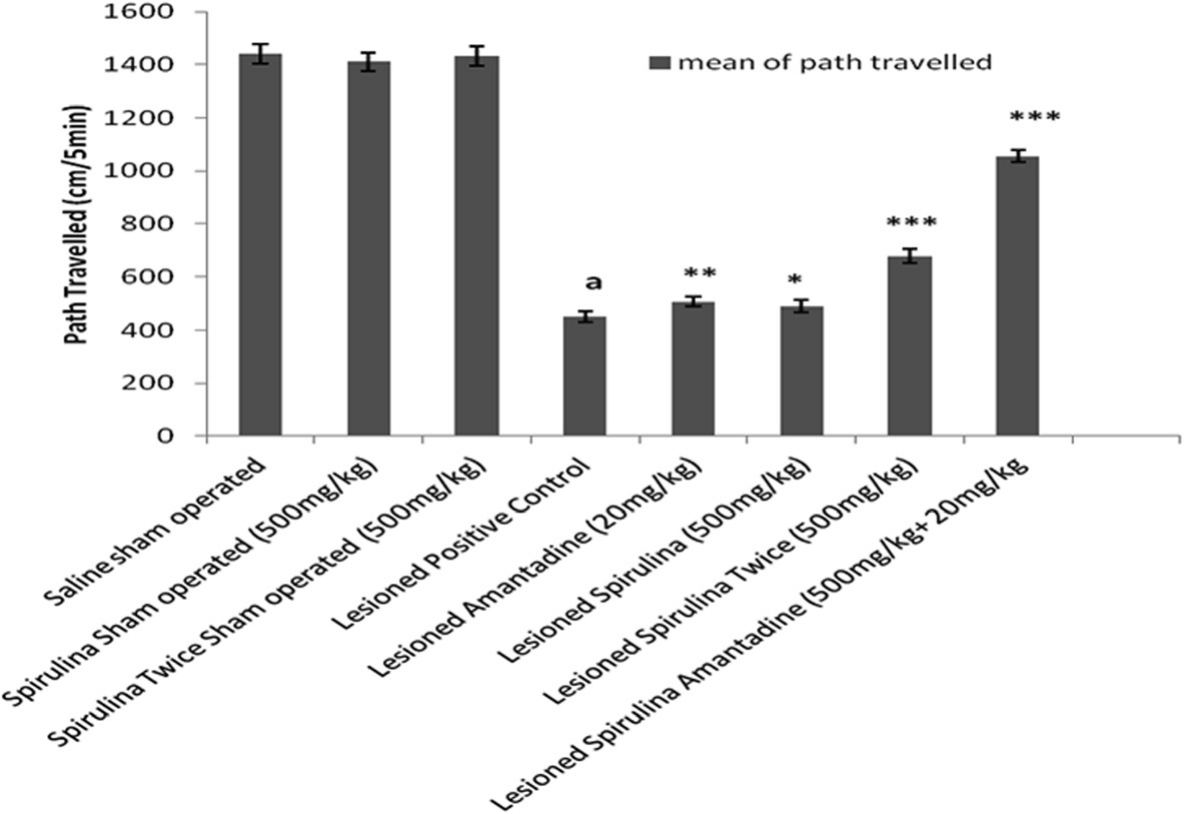
The effect of treatment on body path traveled. Each bar represents the mean ± standard deviation of six animals and the experiments were repeated twice. ^a^ p < 0.001 vs Saline sham operated, *p < 0.05,**p < 0.01,***p < 0.001 VS Lesioned positive control operated

**Fig. 4:**
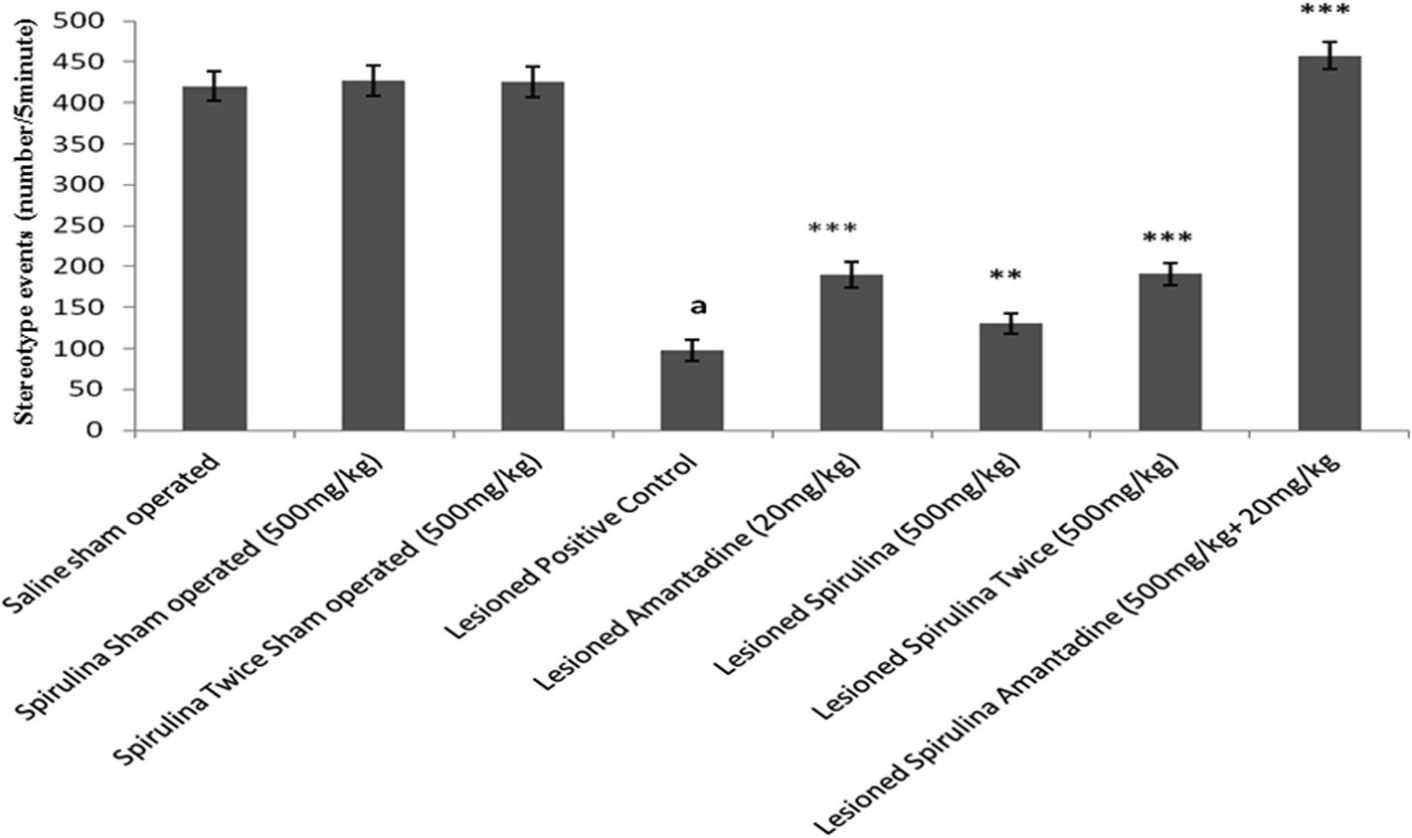
The effect of treatment on stereotype. Each bar represents the mean ± standard deviation of six animals and the experiments were repeated twice. ^a^ p < 0.001 vs Saline sham operated, *p < 0.05,**p < 0.01,***p < 0.001 VS Lesioned positive control operated

Among all lesioned drug treated groups (Fig. 3), spirulina administered twice daily and spirulina with amantadine showed almost 33 and 55 % improvement in the path covered by rats and showed statistically (*p < 0.001*) significant difference when compared with lesioned positive control group declined in path covered (68.78 %). Similar results in case of stereotype events (Fig. 4), treatment of spirulina with amantadine combination exhibited statistically (*p < 0.001*) significant enhancement (78.58 %) when compared with lesioned positive control group (↓76.68 %). Both spirulina treated lesioned groups showed a dose dependent effect statistically (Spirulina 500 mg/kg once daily 24 %, *p < 0.01*; spirulina 500 mg/kg twice daily 48.4 %, *p < 0.001*).

**Fig. 5:**
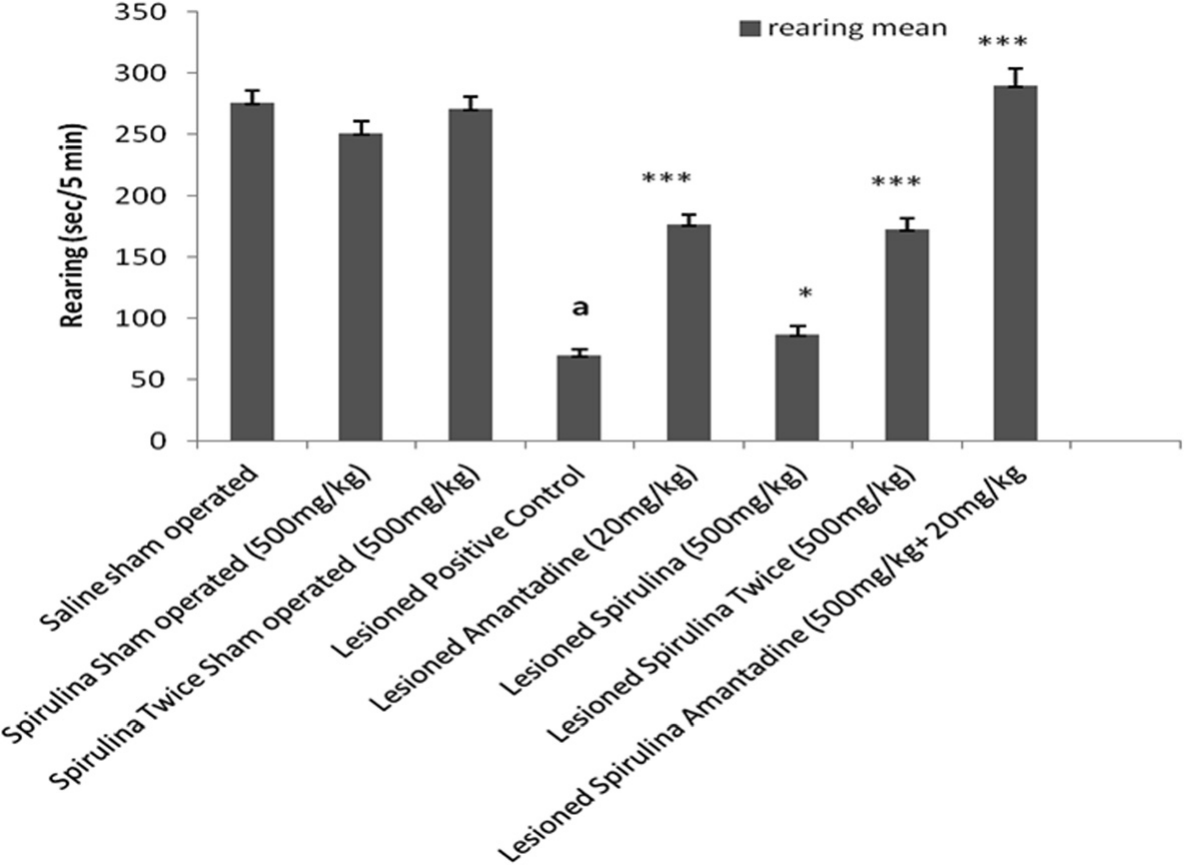
The effect of treatment on rearing. Each bar represents the mean ± standard deviation of six animals and the experiments were repeated twice. ^a^ p < 0.001 vs Saline sham operated, *p < 0.05,**p < 0.01,*** < 0.001 VS Lesioned positive control operated

**Fig. 6:**
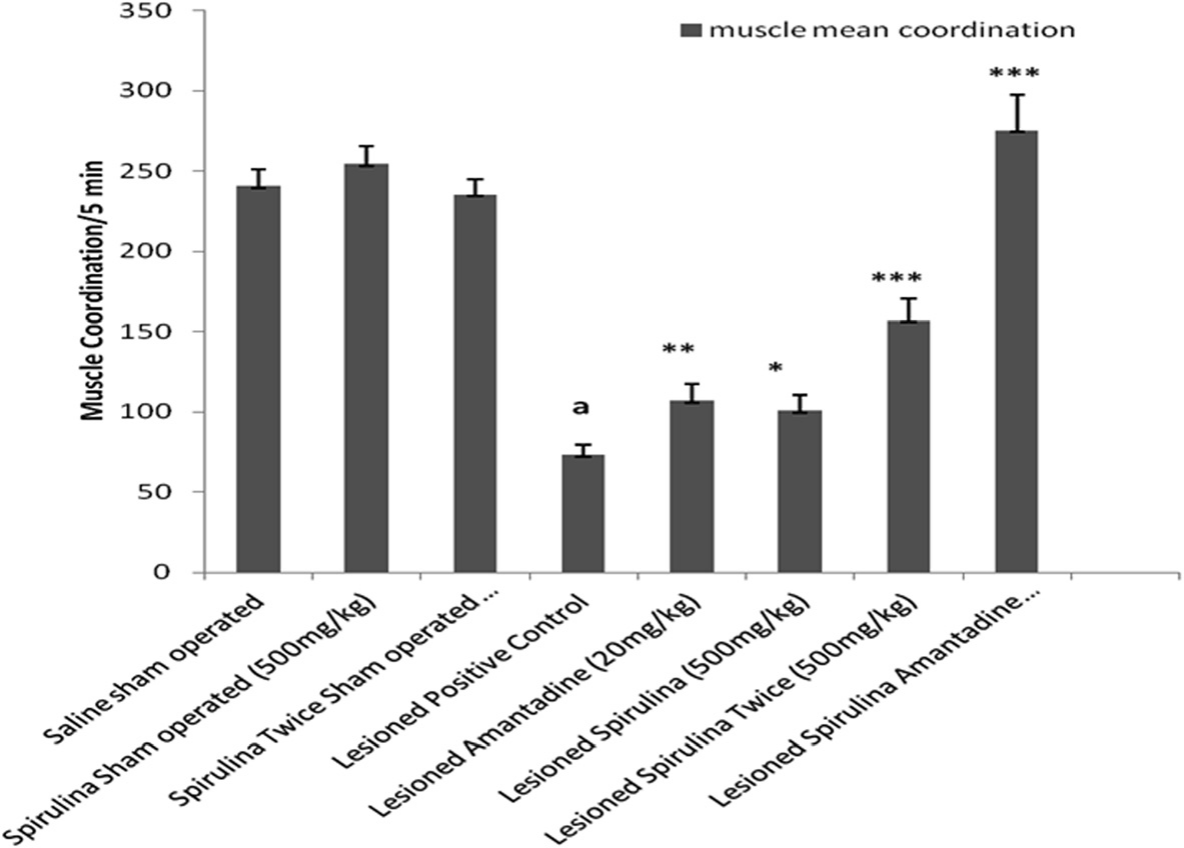
The effect of treatment on muscle coordination. Each bar represents the mean ± standard deviation of six animals and the experiment were repeated twice. ^a^ p < 0.001 vs. Saline sham operated, *p < 0.05,**p < 0.01,***p < 0.001 VS Lesioned positive control operated

Tremendous effect (Fig. 5) was shown by spirulina (500 mg/kg) twice daily revealed three fold improvement (59.67 %) in rearing behavior in comparison with single dose of spirulina (19.81 %). Spirulina with amantadine treated lesioned group developed a 75.96 %, a highly statistically significant (*p < 0.001*) difference. In muscle coordination (Fig. 6), same enhancement (73.51 %) was shown in combination of spirulina with amantadine treated lesioned group than individual drug treatments [Spirulina alone treated lesioned group (27.50 %) and amantadine treated lesioned group (31.78 %)] after compared with lesioned positive control group by 69.98 % declined in muscle movements.

Overall in all behavior activities, spirulina (500 mg/kg, twice daily) and spirulina plus amantadine treated lesioned group confirmed a statistical significantly improvement in the prevention of Parkinsonism behavior. However, no significant effects were shown in any behavior activity in spirulina treated sham operated groups when compared with normal saline sham operated group.

### Effect of Parkinsonism on the content of thiobarbituric acid reactive substances and reduced glutathione and their protection by spirulina

**Fig. 7:**
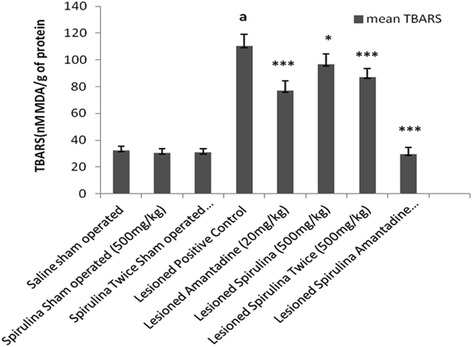
The effect of treatments on thiobarbituric acid reactive substrates. Each bar represents the mean ± standard deviation of six animals and the experiments were repeated twice. ^a^ p < 0.001 vs Saline sham operated, *p < 0.05,**p < 0.01,***p < 0.001 VS Lesioned positive control operated

The level of TBARS in *substantia nigra* was elevated (70.59 %) significantly (*p < 0.001*) in the lesioned positive control group when compared with normal saline sham operated group (Fig. 7). A statistically significant reduction was obtained after chronic administration of spirulina with amantadine treated lesioned group (73.81 %) when compared with lesioned positive control group whereas spirulina (twice daily) treated lesioned group showed only 21.10 % and amantadine alone treated lesioned group exhibited 30.3 %.

**Fig. 8:**
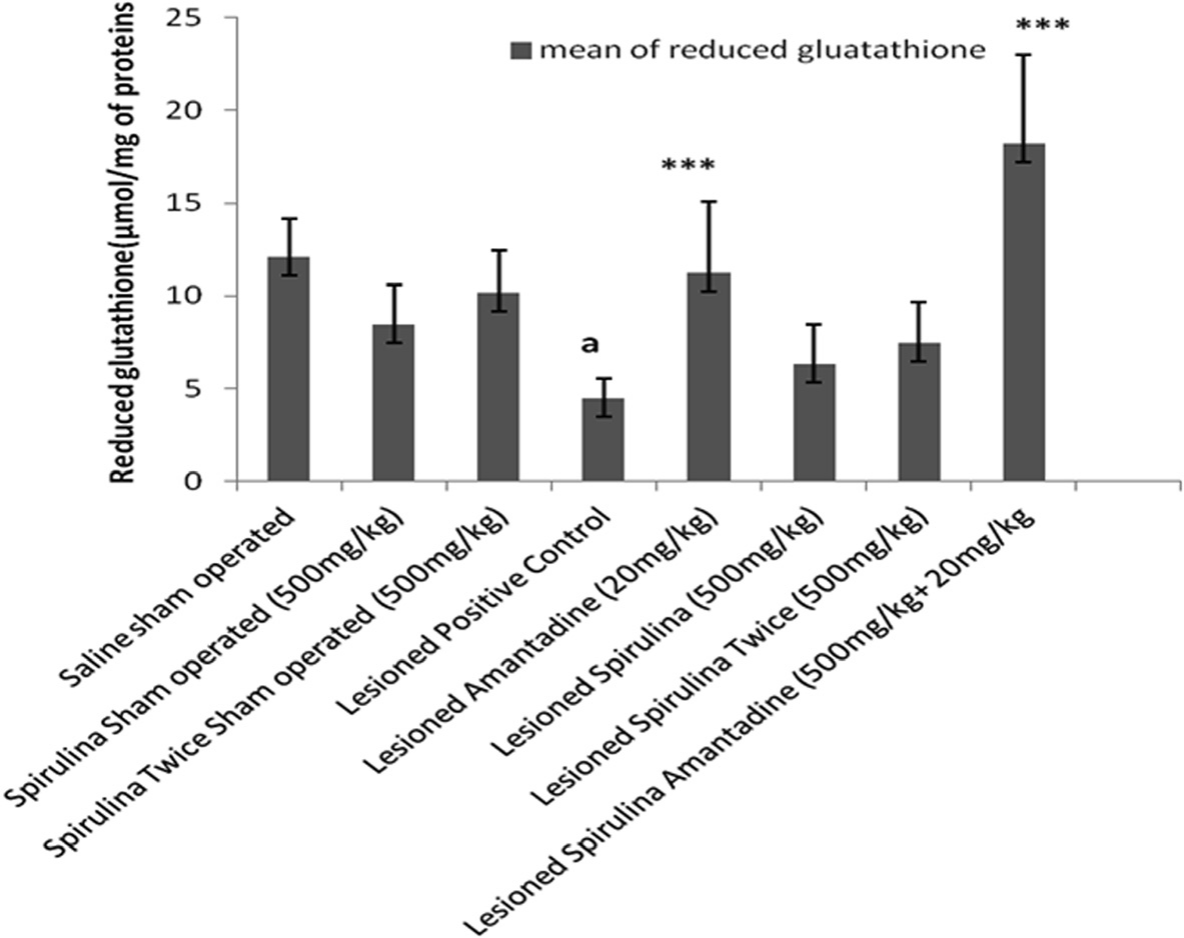
The effect of treatments on reduced glutathione levels. Each bar represents the mean ± standard deviation of six animals and the experiments were repeated twice. ^b^ p < 0.01 vs Saline sham operated, *p < 0.05,**p < 0.01,***p < 0.001 VS Lesioned positive control operated

Similarly, the content of glutathione (Fig. 8) in *substantia nigra* was significantly (*p < 0.01*) depleted (63.14 %) in lesioned positive control group when compared with normal saline sham operated group. In spirulina with amantadine received lesioned group, the glutathione level was restored extremely statistical significant (*p < 0.001*) than spirulina treated lesioned groups. Same in case of dopamine level (Additional file 2: Table S2) showed highly statistical significant increased (*p < 0.001*). Overall, pretreatment of spirulina with amantadine treated lesioned group showed a highly statistical significant effects in all behavior parameters when compared with lesioned positive control group.

## Discussion

Unilateral 6-OHDA-lesioned rats are widely used as a model for Parkinson’s diseases (PD) for the molecular mechanistic study approach involved in neuronal dopaminergic degeneration [29]. Despite an extensive investigation conducted by many researchers, our aim was to find out the effective and easily approach for the treatment of this disorder. A pre and post treatment of spirulina extract alone and in combination of spirulina with amantadine treated lesioned group showed a protective brain response after lesioned and demonstrated a same type of effect in rat behavior which was supported by other studies also [30, 31]. The results of the present study indicated that both pre and post treatment showed a significant extremely effective positive response against 6-OHDA induced abnormal behavior and increased oxidant enzymes in rats. Different behavior activities like body movements, locomotor activity, distance travelled, stereotype events, rearing behavior and muscle coordination (Figs. 1, 2, 3, 4, 5, 6) produced a drastically statistical (*p < 0.001*) significant improvement after treated with spirulina alone and in combination with amantadine. By factorial design (multivariate analysis) at all levels (Additional file 1: Table S1), Overall, there were statistically significant difference (*p < 0.0001*) between groups and within subjects when compared to other positive lesioned treated groups. From our results, it also showed that spirulina (500 mg/kg) potentiates the action of amantadine when treated in combination in 6-OHDA induced damage in rat brain. Similarly, in case of anti-oxidant assays and dopamine level, a combined treatment showed best protective results as a statistical highly significant improvement and restoration of striatal dopamine levels (Additional file 2: Table S2). The content of thiobarbituric acid (Fig. 7) was reduced and glutathione level was increased (Fig. 8).

Various studies have been reported to show contradictory results depending upon various factors like use of different methodologies, strains, geographical regions, environmental factor, indicators of oxidative stress and species of animals. A study conducted by one of the researcher showed that oxidative stress was increased in younger rats rather in all age groups of rats after induced by 6-OHDA due to inability of detoxify free radicals in younger animals than aging animals [32]. Vice versa results were reported through other studies [33]. Another researcher conducted a time course study suggested an alteration of oxidative stress in rats [34, 35].

Brains of PD patient showed low levels of endogenous antioxidants [36]. Antioxidants might be one of the ideal agents to prevent free radical-mediated tissue destruction and inhibit some of the early degenerative events. Natural and endogenous antioxidants such as polyphenols, coenzyme Q10, and vitamins A, C, and E have been proposed as therapeutic agents for preventing and delaying the development of PD [37]. Various epidemiological studies demonstrated a regular consumption of a mediterranean diet (high levels of vitamin C, vitamin E, flavonoids, and carotenoids) can lower the risk of neurological disorders [38–40]. In animal experimental model, chronic usage of blueberry, spinach, or spirulina-enriched diets showed a declines in age-related disorders and restores the dopamine levels in the striatum [41–46].

In our study, at 48^th^ day, spirulina extract alone treated lesioned group and in combination with amantadine showed a marvelous antioxidant activity through various antioxidant assays however, dopamine level could not reach near normal level, in spirulina alone (500 mg/kg once daily and twice daily) treated lesioned group when compared with amantadine combination. A similar study was supported to show anti-inflammatory and anti-oxidant properties with blueberry and spirulina enriched diet, improved a recovery at the nerve terminal rather than cellular level [17]. After 1 month the recovery of striatal TH-positive nerve fibers were increased while a loss of dopamine neurons was slightly improved. This type of effect is may be due to sprouting from non-injured dopamine neurons rather from regenerating rescued dopamine neurons or up regulation of lost TH synthesis which was reported by Stromberg [17]. Furthermore, it was also reported that, spirulina blocked the cyclooxygenase pathway which showed significant regeneration of TH-positive nerve fibers [47]. The probable mechanism responsible for this effect is may be due to the presence of phycocyanin, phycocyanobilin and beta carotene, as these are reported to have strong anticyclooxygenase-2 and antioxidant activities [10, 48]. Another study also reported that spirulina supplementation decreases the lipid peroxidation and increased the levels of reduced glutathione in humans [49]. Extracts of spirulina have antioxidant activity both *in vivo* and *in vitro*. Similar results were found with spirulina treated on rats against the neuronal loss induced by α-synuclein [19].

Another potential constituent present in spirulina is gamma-linolenic acid which decreased the lipid peroxidation [50]. Indeed, it has been found that an increased ratio of gamma-linolenic acid to arachidonic acid is capable of attenuating the biosynthesis of arachidonic acid metabolites (i.e., prostaglandins, leukotrienes, and platelet-activating factor) and exerts an anti-inflammatory effect [50]. Decreased inflammation via this route might have decreased the production of superoxide, hydrogen peroxide, and hypochlorous acid by the activated neutrophils leading to less lipid peroxidation after spirulina supplementation. There are many active components present in spirulina which may show an insight stories about excellent protectively in various disorders. Many of spirulina constituents may interact with microglia to reduce microglia activation. The reason for this effect is still unknown. Excellent synergistic neuroprotective effect was shown by spirulina with amantadine combination and neuroprotective effect of amantadine is may be due to formation of conjugates/derivatives which was also reported in earlier studies [51, 52]. However, pharmacokinetic interaction between spirulina and amantadine will provide more insight into this beneficial interaction.

Although disease-modifying therapies have not yet found their way to clinical practice, a substantial number of compounds have been identified as neuroprotective in preclinical studies. Since most physicians rarely access or appraise scientific evidence directly from research results, most neurologists have overlooked preclinical improvements, resulting in lowered expectations and misplaced doubts about neuroprotection. Spirulina supplementation has been shown to offer significant protection against a number of environmental toxicants, chemical or drug-induced oxidative stress and inflammation.

Many patients on amantadine therapy are mandatory to take antioxidants simultaneously. Hence, the results of this study have direct relevance clinically. Though, the results of this study can be extrapolated to beneficial effects of combination of amantadine with other antioxidants, we recommend that such combinations should only be used only after thorough pharmacokinetic and pharmacodynamic interaction studies. There are multi path physiology are involved which can lead to Parkinson’s disease such as cyclooxygenase pathway, TNF-α and cholinergic action. It is very difficult to predict the anti Parkinsonism effect at pharmacological level unless until the molecular levels studies are conducted.

## Conclusion

Finally, it is concluded that, pretreatment with spirulina showing anti-parkinsonism effect on behavior and anti-oxidant parameters on 6-OHDA induced dopaminergic damage rats. Spirulina also potentiates the anti-parkinsonian effect of amantadine when administered in combination. The neurodegenerative disorder is well known in aging related problems but now a day’s many of the researchers reported that chronic condition of type 2 diabetes mellitus induces neurodegenerative disorders like Parkinsonism and Alzheimer diseases. So, this study is preliminary study on Parkinsonism rat model and spirulina is an added advantage to Parkinsonism patients when given with amantadine in chronic type 2 diabetes induced neurodegenerative disorder especially in Parkinsonism disorder in future studies.

## Additional files

Additional file 1: Table S1. Effect of treatments on body rotations between groups and within groups with multivariate analysis. (DOC 106 kb) Additional file 2: Table S2. Effect on dopamine levels. (DOC 32 kb)
