# Erratum to: Neuroprotective effect of *Spirulina fusiform* and amantadine in the 6-OHDA induced Parkinsonism in rats

**DOI:** 10.1186/s12906-015-0855-5

**Published:** 2015-09-09

**Authors:** I. Chattopadhyaya, Sumeet Gupta, Asad Mohammed, N. Mushtaq, S. Chauhan, Saikat Ghosh

**Affiliations:** Department of Pharmacology, M. M. College of Pharmacy, M. M. University, Mullana, (Ambala), Haryana India; College of Applied Medical Sciences, Shaqra University, Shaqra, Saudi Arabia; FDD-NDDS Lipid Research Group, Sun Pharma Advance Research Centre, Vadodra, Gujarat India

Unfortunately, the original version of this article [1] contained an error. The spelling of the author [Saikant Ghosh] name was incorrect. The spelling of the name has been corrected in the original article and is also included correctly below.

Correct version is Saikat Ghosh.
