# Supplementary material for: Neuroprotective effect of Spirulina fusiform and amantadine in the 6-OHDA induced Parkinsonism in rats
Source: BMC Complement Altern Med. 2015 Aug 25;15:296. doi: 10.1186/s12906-015-0815-0 (PMC4548915; doi:10.1186/s12906-015-0815-0)
Supplement: Additional file 1: Table S1. — Effect of treatments on body rotations between groups and within groups with multivariate analysis. (DOC 106 kb) [file 12906_2015_815_MOESM1_ESM.doc]

**Table 1: Effect of treatments on body rotations between groups and within groups with** multivariate analysis

| **Amphetamine** | | | | | | | | | | | | | | | | | |
| --- | --- | --- | --- | --- | --- | --- | --- | --- | --- | --- | --- | --- | --- | --- | --- | --- | --- |
| **Groups** | **Group 1** | | | **Group 2** | | | **Group 3** | **Group 4** | | | **Group 5** | | **Group 6** | | **Group 7** | | **Group 8** |
| **Mean ± SD** | 4.21 ± 0.710 | | | 5.22 ± 0.772 | | | 3.64 ± 0.622 | 1.43 ± 2.93 | | | 7.27 ± 1.48 | | 1.06 ± 1.49 | | 9.46 ± 2.87 | | 6.17 ± 1.65 |
| **95% confidence interval**  **Lower bound- upper bound** | 3.80-4.62 | | | 4.77-5.66 | | | 3.28-4.0 | 12.63-16.02 | | | 6.42-8.13 | | 9.77-11.50 | | 7.80-11.12 | | 5.22-7.13 |
| **Apomorphine** | | | | | | | | | | | | | | | | | |
| **Mean ± SD** | | 3.87 ± 1.61 | | | 7.79 ± 1.79 | | 6.24 ± 1.14 | 1.96 ± 4.27 | | | 9.49 ± 3.70 | | 1.24 ± 3.64 | | 9.60 ± 2.07 | | 4.36 ± 0.578 |
| **95% confidence interval**  **Lower bound- upper bound** | | 2.93-4.80 | | | 6.75-8.82 | | 5.58-6.90 | 17.1-22.08 | | | 7.35-11.63 | | 10.38-14.59 | | 8.4-10.79 | | 4.03-4.69 |
| **ANOVA** | | | | | | | | | | | | | | | | | |
| **Amphetamine** | | | Between groups | | | Sum of squares | | | df | | | Mean square | | F | | *P value* | |
|  |  |  |  |  |  | 1301.82 | | | 7 | | | 185.91 | | 58.21 | | P<0.0001 | |
|  |  |  | Within groups | | | 332.12 | | | 104 | | | 3.19 | |  | |  | |
| **Apomorphine** | | | Between groups | | | 2547.90 | | | 7 | | | 363.99 | | 51.04 | | P<0.0001 | |
|  |  |  | Within groups | | | 741.58 | | | 104 | | | 7.131 | |  | |  | |
| **Multivariate tests** | | | | | | | | | | | | | | | | | |
| **Effect** | | | | | | **Value** | | | | **F** | | **Hypothesis df** | | **Error df** | | **Sig.** | |
| **Intercept** | | | **Pillai's Trace** | | | .963 | | | | 1.336E3^a^ | | 2.000 | | 103.000 | | .000 | |
|  | | | **Wilks' Lambda** | | | .037 | | | | 1.336E3^a^ | | 2.000 | | 103.000 | | .000 | |
|  | | | **Hotelling's Trace** | | | 25.934 | | | | 1.336E3^a^ | | 2.000 | | 103.000 | | .000 | |
|  | | | **Roy's Largest Root** | | | 25.934 | | | | 1.336E3^a^ | | 2.000 | | 103.000 | | .000 | |
| **Groups** | | | **Pillai's Trace** | | | 1.111 | | | | 18.553 | | 14.000 | | 208.000 | | .000 | |
|  | | | **Wilks' Lambda** | | | .111 | | | | 29.425^a^ | | 14.000 | | 206.000 | | .000 | |
|  | | | **Hotelling's Trace** | | | 6.003 | | | | 43.737 | | 14.000 | | 204.000 | | .000 | |
|  | | | **Roy's Largest Root** | | | 5.650 | | | | 83.942^b^ | | 7.000 | | 104.000 | | .000 | |

Post hoc bonferrri

| **Amphetamine** | **Groups** | | ***P value*** | **Apomorphine** | **Groups** | | ***P value*** |
| --- | --- | --- | --- | --- | --- | --- | --- |
|  | Group 1 | Group 2 | ns |  | Group 1 | Group 2 | 0.005 |
|  |  | Group 3 | ns |  |  | Group 3 | ns |
|  |  | Group 4 | .000 |  |  | Group 4 | .000 |
|  |  | Group 5 | .000 |  |  | Group 5 | .000 |
|  |  | Group 6 | .000 |  |  | Group 6 | .000 |
|  |  | Group 7 | .000 |  |  | Group 7 | .000 |
|  |  | Group 8 | .124 |  |  | Group 8 | ns |
|  | Group 2 | Group 1 | ns |  | Group 2 | Group 1 | .005 |
|  |  | Group 3 | ns |  |  | Group 3 | ns |
|  |  | Group 4 | .000 |  |  | Group 4 | .000 |
|  |  | Group 5 | ns |  |  | Group 5 | ns |
|  |  | Group 6 | 0.000 |  |  | Group 6 | .000 |
|  |  | Group 7 | 0.000 |  |  | Group 7 | ns |
|  |  | Group 8 | ns |  |  | Group 8 | .027 |
|  | Group 3 | Group 1 | ns |  | Group 3 | Group 1 | ns |
|  |  | Group 2 | ns |  |  | Group 2 | ns |
|  |  | Group 4 | .000 |  |  | Group 4 | .000 |
|  |  | Group 5 | .000 |  |  | Group 5 | .048 |
|  |  | Group 6 | .000 |  |  | Group 6 | .000 |
|  |  | Group 7 | .000 |  |  | Group 7 | .034 |
|  |  | Group 8 | .008 |  |  | Group 8 | ns |
|  | Group 4 | Group 1 | .000 |  | Group 4 | Group 1 | .000 |
|  |  | Group 2 | .000 |  |  | Group 2 | .000 |
|  |  | Group 3 | .000 |  |  | Group 3 | .000 |
|  |  | Group 5 | .000 |  |  | Group 5 | .000 |
|  |  | Group 6 | .000 |  |  | Group 6 | .000 |
|  |  | Group 7 | .000 |  |  | Group 7 | .000 |
|  |  | Group 8 | .000 |  |  | Group 8 | .000 |
|  | Group 5 | Group 1 | .000 |  | Group 5 | Group 1 | .000 |
|  |  | Group 2 | ns |  |  | Group 2 | ns |
|  |  | Group 3 | .000 |  |  | Group 3 | .048 |
|  |  | Group 4 | .000 |  |  | Group 4 | .000 |
|  |  | Group 6 | .000 |  |  | Group 6 | .105 |
|  |  | Group 7 | 0.46 |  |  | Group 7 | ns |
|  |  | Group 8 | ns |  |  | Group 8 | .000 |
|  | Group 6 | Group 1 | .000 |  | Group 6 | Group 1 | .000 |
|  |  | Group 2 | .000 |  |  | Group 2 | .000 |
|  |  | Group 3 | .000 |  |  | Group 3 | .000 |
|  |  | Group 4 | .000 |  |  | Group 4 | .000 |
|  |  | Group 5 | .000 |  |  | Group 5 | .105 |
|  |  | Group 7 | ns |  |  | Group 7 | .144 |
|  |  | Group 8 | .000 |  |  | Group 8 | .000 |
|  | Group 7 | Group 1 | .000 |  | Group 7 | Group 1 | .000 |
|  |  | Group 2 | .000 |  |  | Group 2 | ns |
|  |  | Group 3 | .000 |  |  | Group 3 | .034 |
|  |  | Group 4 | .000 |  |  | Group 4 | .000 |
|  |  | Group 5 | .046 |  |  | Group 5 | ns |
|  |  | Group 6 | ns |  |  | Group 6 | .144 |
|  |  | Group 8 | .000 |  |  | Group 8 | .000 |
|  | Group 8 | Group 1 | ns |  | Group 8 | Group 1 | ns |
|  |  | Group 2 | ns |  |  | Group 2 | .027 |
|  |  | Group 3 | .008 |  |  | Group 3 | ns |
|  |  | Group 4 | .000 |  |  | Group 4 | .000 |
|  |  | Group 5 | ns |  |  | Group 5 | .000 |
|  |  | Group 6 | .000 |  |  | Group 6 | .000 |
|  |  | Group 7 | .000 |  |  | Group 7 | .000 |
