# Supplementary material for: Neuroprotective effect of Spirulina fusiform and amantadine in the 6-OHDA induced Parkinsonism in rats
Source: BMC Complement Altern Med. 2015 Aug 25;15:296. doi: 10.1186/s12906-015-0815-0 (PMC4548915; doi:10.1186/s12906-015-0815-0)
Supplement: Additional file 2: Table S2. — Effect on dopamine levels. (DOC 32 kb) [file 12906_2015_815_MOESM2_ESM.doc]

**Table 1: Effect on dopamine levels.**

| **S. no** | **Groups** | **Dopamine levels (ng/g)** |
| --- | --- | --- |
| **1** | Sham operated Normal (saline) | 7.21 ± 0.74 |
| **2** | Sham operated + Spirulina (500 mg/kg b.w/day per oral) | 6.36 ± 0.46 |
| **3** | Sham operated + Spirulina (500 mg/kg b.w/twice a day per oral) | 6.91 ± 0.61 |
| **4** | Lesioned Positive control group | 1.56 ± 0.33^a^ |
| **5** | Lesioned + Amantadine 20mg/kg orally) | 2.87 ± 0.76** |
| **6** | Lesioned + Spirulina (500 mg/kg b.w/day per oral) | 1.89 ± 0.54 |
| **7** | Lesioned + Spirulina (500 mg/kg b.w/twice a day per oral) | 1.98 ± 0.35 |
| **8** | Lesioned + Spirulina (500 mg/kg b.w/day per oral) + amantadine 20mg/kg orally) | 7.03 ± 0.61*** |

Over all P value *p<0.0001*

*F value* 106.8

Statistical analysis of data was carried by one-way ANOVA followed by Tukey’s Multiple Range Test. The values are Mean ± SD for each group (n=6) and the experiments were repeated twice. ^a^*p<0.001 vs Saline sham operated, ***p<0.05,****p<0.01,*****p<0.001 VS Lesioned positive control.*
